# Supplementary material for: Staphylococcus aureus sacculus mediates activities of M23 hydrolases
Source: Nat Commun. 2023 Oct 23;14:6706. doi: 10.1038/s41467-023-42506-w (PMC10593780; doi:10.1038/s41467-023-42506-w)
Supplement: Supplementary file 1 — Supplementary Information [file 41467_2023_42506_MOESM1_ESM.pdf]

Supplementary information

Staphylococcus aureus sacculus mediates activities of M23 hydrolases

Razew[1,2,3] Alicja, Laguri[1] Cedric, Vallet[1] Alicia, Bougault[1] Catherine, Kaus-Drobek[3] Magdalena, Sabala[2,3] Izabela* & Simorre[1] Jean-Pierre*

[1]Universite Grenoble Alpes, CNRS, CEA, Institut de Biologie Structurale, 71 avenue des Martyrs-CS10090, Grenoble cedex 9 38044, France

[2]International Institute of Molecular and Cell Biology in Warsaw, 4 Ks. Trojdena Street, 02-109, Warsaw, Poland

[3]Laboratory of Protein Engineering, Mossakowski Medical Research Institute, Polish Academy of Sciences, 5 Pawinskiego Street, 02-106, Warsaw, Poland

Correspondence: isabala@imdik.pan.pl, jean-pierre.simorre@ibs.fr

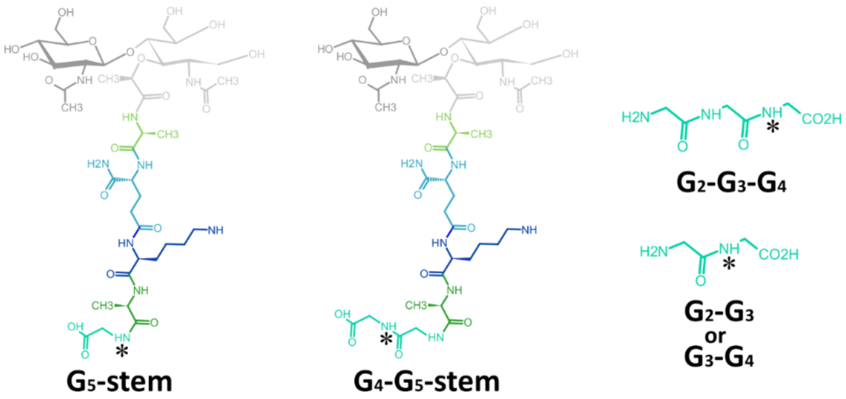

Supplementary Figure 1. Chemical structure of the non cross-linked peptidoglycans and oligoglycin peptides generated by treatment of muropeptide multimers with studied hydrolytic enzymes. NH groups producing NMR signals characteristic of cleavage are marked with an asterisk. Schemes were done using ACD/ChemSketch® software. Amino acids and sugars were color-coded as in Fig. 1a in main text.

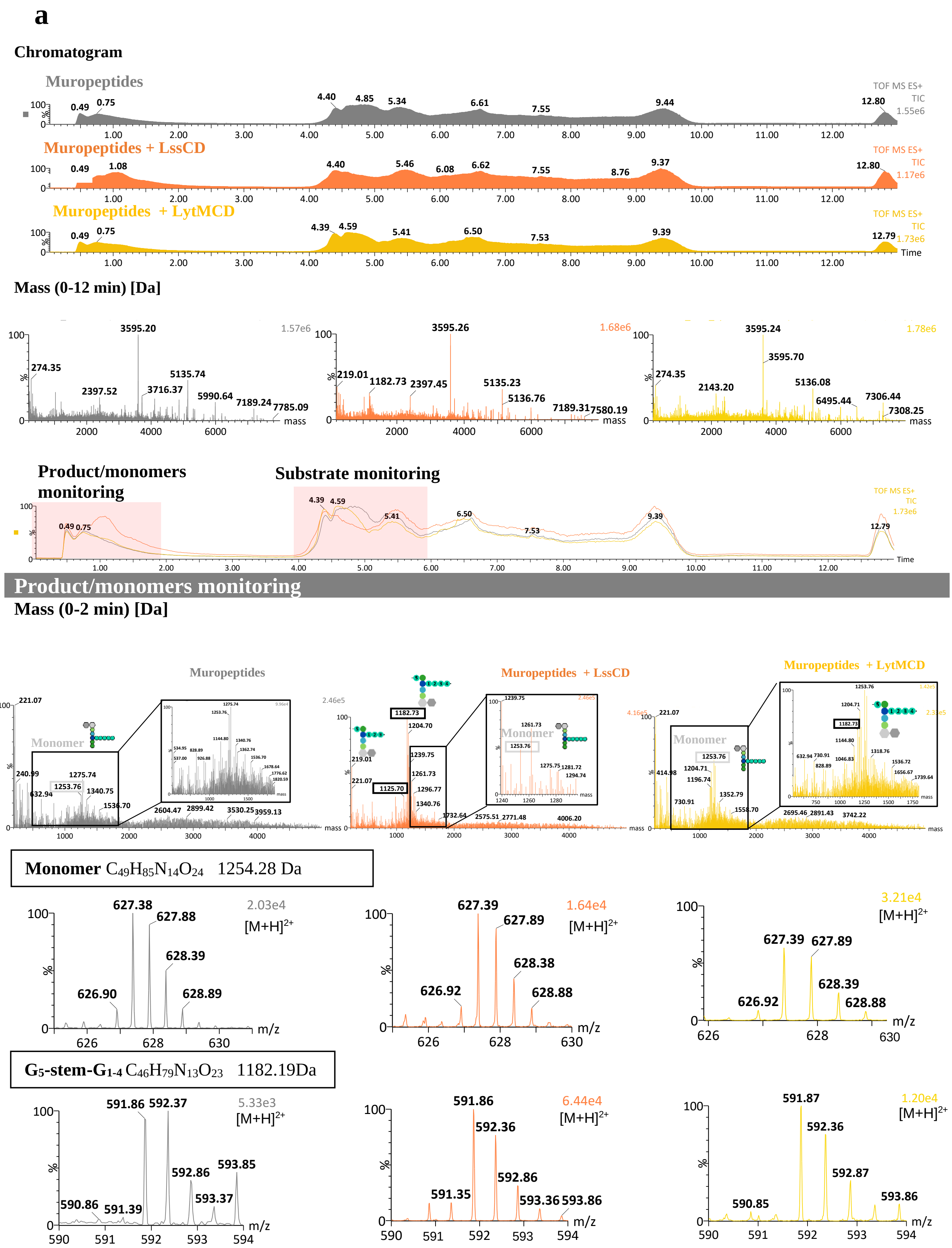
a
Chromatogram
Muropeptides
Muropeptides + LssCD
Muropeptides + LytMCD
TOF MS ES+
TIC
1.55e6
1.17e6
1.73e6
Time
Mass (0-12 min) [Da]
3595.20
3595.26
3595.24
Product/monomers monitoring
Substrate monitoring
Product/monomers monitoring
Mass (0-2 min) [Da]
Monomer
Monomer $C_{49}H_{85}N_{14}O_{24}$ 1254.28 Da
627.38
627.39
[M+H]$^{2+}$
2.03e4
1.64e4
3.21e4
G5-stem-G1-4 $C_{46}H_{79}N_{13}O_{23}$ 1182.19Da
591.86
591.87
5.33e3
6.44e4
1.20e4
m/z

G5-stem-G1-3 $C_{44}H_{76}N_{12}O_{22}$ 1125.14 Da

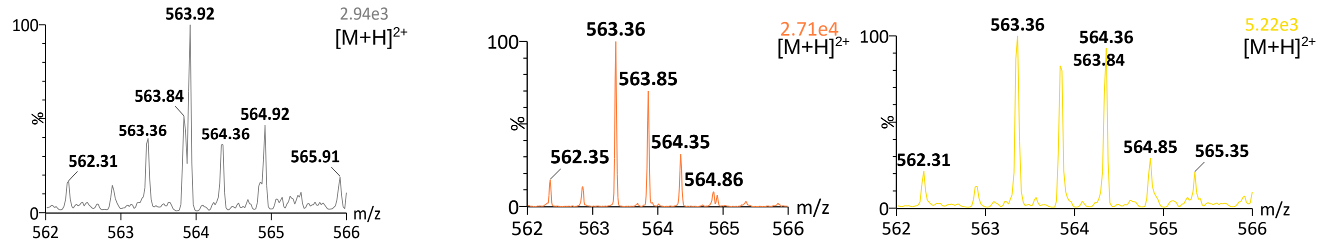

G5-stem-G1-2 $C_{42}H_{73}N_{11}O_{21}$ 1068.09 Da

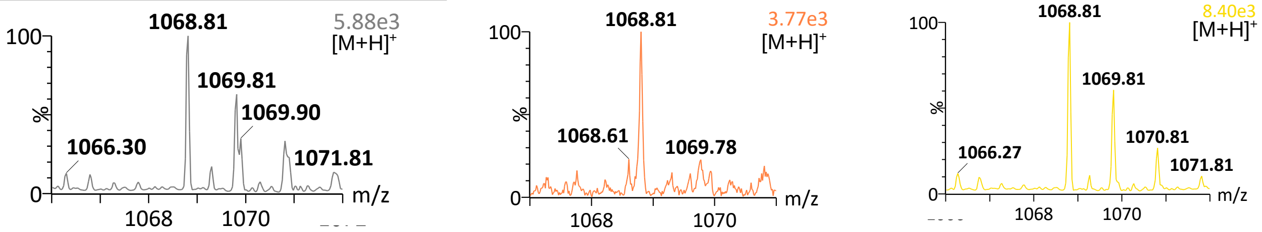

Substrate monitoring

Mass (4-6 min) [Da]

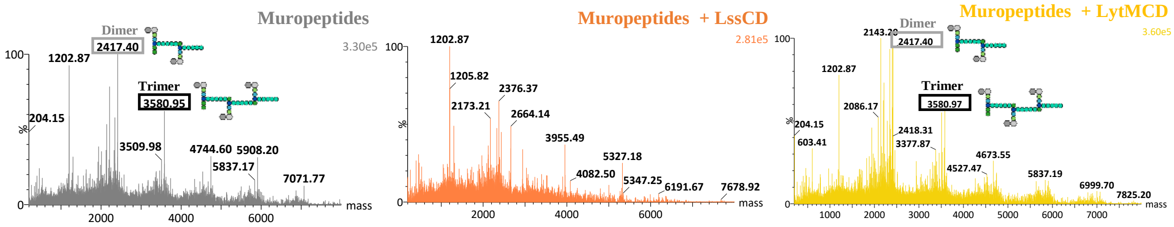

m/z of detected muropeptides

Trimer $C_{141}H_{239}N_{40}O_{68}$ 3582.62 Da

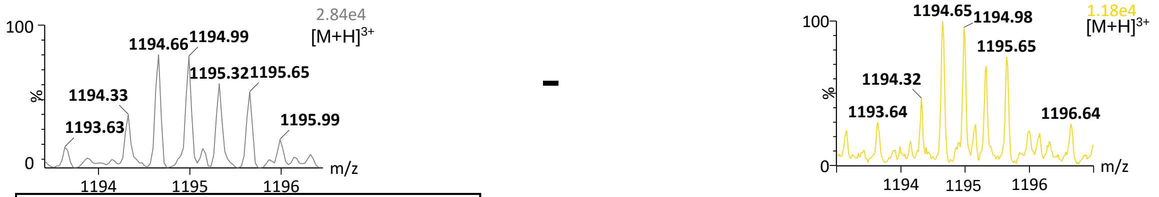

Dimer $C_{95}H_{162}N_{27}O_{46}$ 2418.45 Da

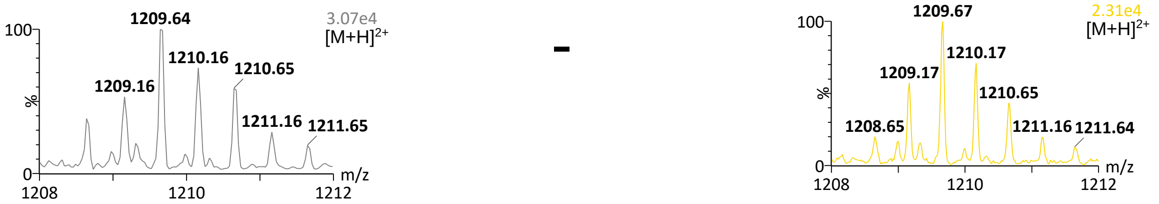

b

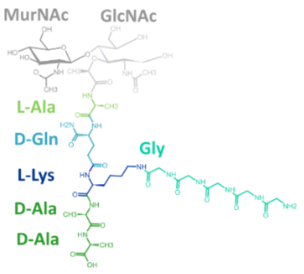

Muropeptide monomer

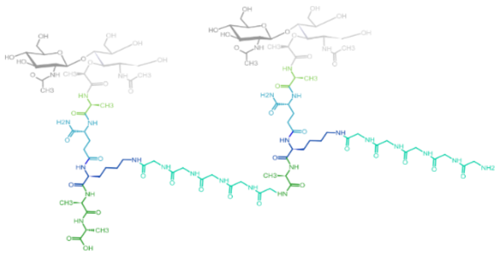

Muropeptide dimer

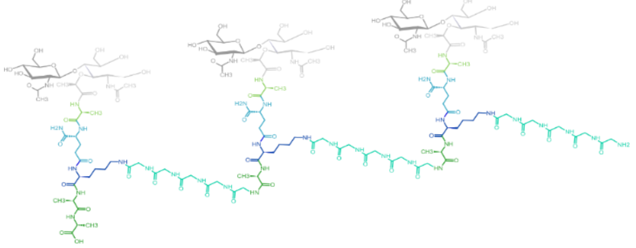

Muropeptide trimer

Supplementary Figure 2. Analysis of muropeptide digestion by Lss and LytM catalytic domains using UPLC-MS.

a, UPLC chromatograms, m/z and mass spectra of *S. aureus* TF5303 muropeptides obtained after mutanolysin digestion (grey) and further processing with LssCD or LytMCD (orange and yellow, respectively). Entire chromatogram was searched for the masses corresponding to hypothetical substrates of the enzymes (monomer, dimer, trimer) and reaction products. Selected ranges (0-2 min and 4-6 min) were investigated to underlie differences between spectra. In all presented mass and m/z graphs, vertical axis represents relative intensity expressed in percent. m/z for selected muropeptides were reported, the value in right bottom corner indicates the intensity of the highest peak in the presented spectrum. m/z of dimer and trimer were not reported for LssCD due to their low signal. Presented mass spectra are deconvoluted, while zoomed ones are raw spectra. Chemical formula and calculated theoretical exact mass of the monitored species was reported in frames.

b, Chemical structures of the muropeptides identified in analysed samples. Schemes and calculation of the masses were done using ACD/ChemSketch® software.

Supplementary Table 1 Intensities of muropeptide digestion products identified from data in Figure S2a in spectrum range between 0 and 2 min. MS intensities of sodium and potassium adducts for each monitored species were included in the presented values.

**Muropeptide product**	**Muropeptides**	**+ LssCD**	**+ LytMCD**
G_5-stem-G_{1-4}	1.16×10^5	9.00×10^5	3.49×10^5
G_5-stem-G_{1-3}	2.88×10^4	1.64×10^5	Not detected
G_5-stem-G_{1-2}	7.71×10^4	1.99×10^5	1.23×10^5
G_5-stem-G_1	Not detected	2.54×10^3*	Not detected

*m/z peak indistinguishable from the noise

Supplementary Table 2 Intensities of muropeptides identified from data in Figure S2a in spectrum range between 0 and 2 min (monomer), and 4 and 6 min. MS intensities of sodium and potassium adducts for each monitored species were included in the presented values.

**Muropeptide multimer**	**Muropeptides**	**+ LssCD**	**+ LytMCD**
Monomer	2.26×10^5	2.06×10^5	3.09×10^5
Dimer	4.66×10^5	1.07×10^5	5.13×10^5
Trimer	2.94×10^5	Not detected	3.07×10^5

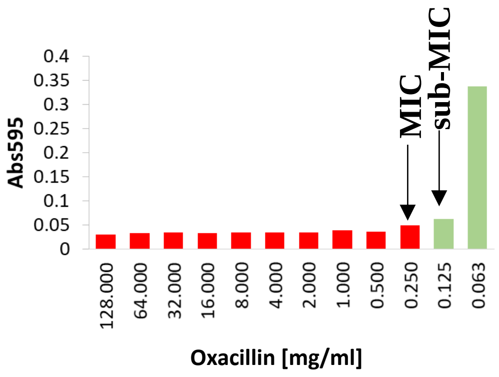

Supplementary Figure 3. Minimal Inhibitory Concentration (MIC) assay of *S. aureus* TF5303 sensitivity to oxacillin.

The sub-MIC concentration of oxacillin marked with arrow was used in the lytic assays. Abs595 – absorbance measured at 595 nm.

a

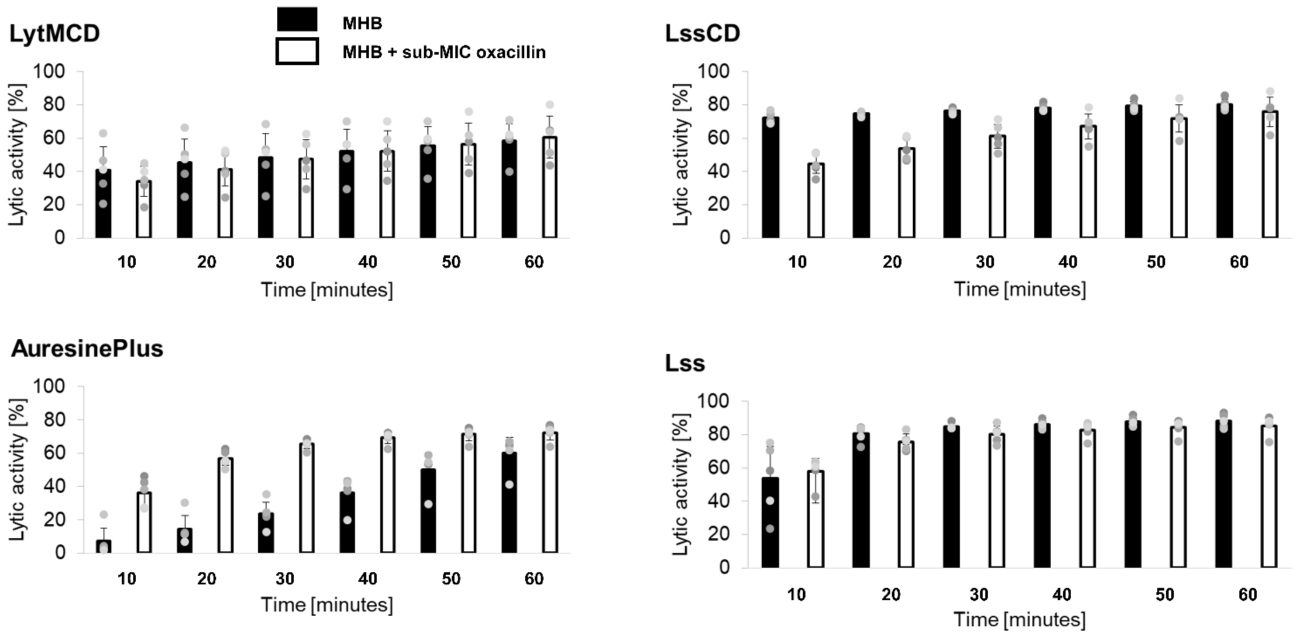

b

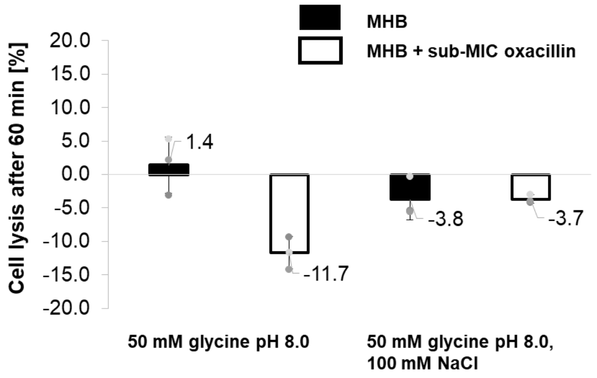

Supplementary Figure 4. Turbidity reduction assay on *S. aureus* TF5303 with regular or decreased crosslinking.

a, Lytic activity of the enzymes against *S. aureus* TF5303 with regular or decreased crosslinking at selected time points. Activity was monitored as turbidity reduction corresponding to absorbance 595 nm. Reaction was conducted in optimal conditions, namely 50 mM glycine buffer pH 8.0 for LytMCD and LssCD, and 50 mM glycine, 100 mM NaCl buffer pH 8.0 for AuresinePlus and Lss. The results are presented in relative to the sample of bacteria suspended in the buffer without enzyme addition. The experiments were performed in five biological and three technical replicates. Source data are provided as a Source Data file.

b, Monitoring of the cell lysis in no-enzyme control after 60 min. Lysis of cells was observed in two vehicles used for the turbidity reduction assay – 50 mM glycine buffer pH 8.0 and 50 mM glycine, 100 mM NaCl buffer pH 8.0. The experiments were performed in three biological and three technical replicates. Abbreviation: MHB - Mueller Hinton Broth. Source data are provided as a Source Data file.

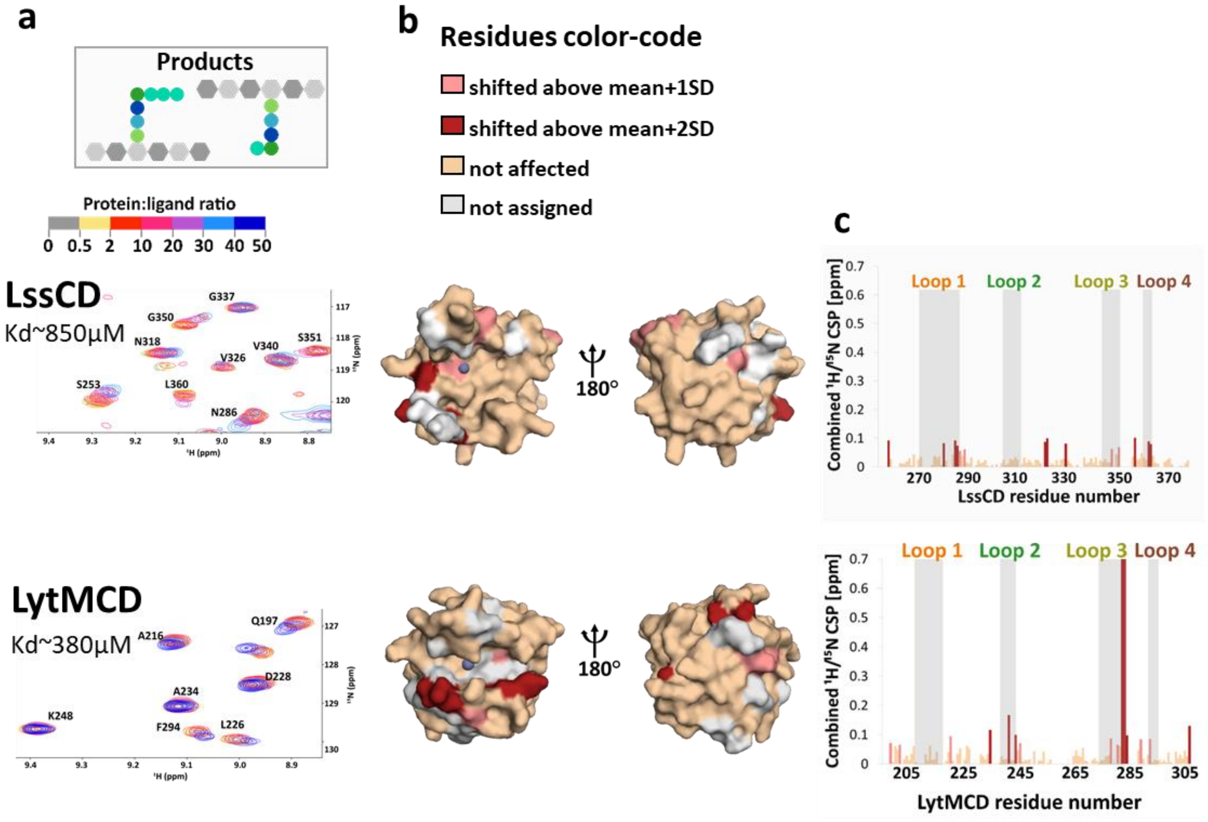

Supplementary Figure 5. LytMCD and LssCD interact with the reaction product.

a, Region of the $^{1}H,^{15}N$-BEST-TROSY spectra of ^{15}N-labelled LssCD (top) and LytMCD (bottom) recorded at 25°C and pH 7.0. The experiment was conducted before (grey) and after addition of Lss endopeptidase reaction products at increasing ligand-to-protein ratios (colour scale from pale yellow to blue). A schematic representation of the M23 endopeptidase reaction product structure is presented. It contains oligosaccharidic PG fragments with disrupted cross-links. The presented structures correspond to the PDB deposits (4ZYB for LyMCD and 5NMY for LssCD).

b, CSP superior to 1 standard deviation (>0.05 ppm for LssCD; >0.06 ppm for LytMCD coloured in salmon) or 2 standard deviations (>0.07 ppm for LssCD; >0.10 ppm for LytMCD coloured in crimson) are displayed on the surface representation of respective protein. Unaffected protein regions were coloured wheat and unassigned white. The left panel corresponds to the same orientation as the right one but rotate by 180°. The zinc was presented as a blue-grey sphere.

c, Combined ^{1}H and ^{15}N chemical shift perturbations calculated for each residue calculated from the data displayed in panel **b**, as the weighted-average distance between the resonance position of the free form of LytM or Lss catalytic domains and its equivalent position in the complex with product measured at the ratio of 1:46 and 1:40 respectively. Bars were colour-coded with CSP amplitudes as in in panel **b**. The loop regions are highlighted in grey.

Supplementary Table 3. Chemical shift perturbations detected for LssCD and LytMCD upon titration with a substrate analogue or product. For the first one, CSP superior to 1 standard deviation (SD > 0.25 ppm for LssCD; > 0.13 ppm for LytMCD) or 2 standard deviations (> 0.38 ppm for LssCD; > 0.20 ppm for LytMCD) were coloured salmon and crimson, respectively. For product, CSP superior to 1 standard deviation (> 0.05 ppm for LssCD; >0.06 ppm for LytMCD) or 2 standard deviations (> 0.07 ppm for LssCD; > 0.10 ppm for LytMCD), and for sacculus, CSP superior to 1 standard deviation (> 0.234 ppm for LssCD; > 0.164 ppm for LytMCD) or 2 standard deviations (> 0.352 ppm for LssCD; > 0.230 ppm for LytMCD) were coloured as for the substrate analogue.

	Residue ID					
	**LssCD**			**LytMCD**		
	Substrate analogue	Product	Sacculus	Substrate analogue	Product	Sacculus
Description	Dissacharide stem-peptides crosslinked by G_4S peptides	Multisaccharide stem-peptide with glycine or diglycine at D-Ala	Fully polymerised sacculus crosslinked by G_4S peptides	Dissacharide stem-peptides crosslinked by G_4S peptides	Multisaccharide stem-peptide with glycine or diglycine at D-Ala	Fully polymerised sacculus crosslinked by G_4S peptides
K_{off} [µM]	71	850	-	78	380	-
**LOOP 1**	**267G** **272G** **274N** **277M** **279Y** **282D**	**277M** **282D** **283F**	**272G** **273I 275G** **277M 278H**	-	-	-
**LOOP 2**	-	-	**308G**	**241G**	**238N 241G**	**235G 239Y** **240G 241G** **242G**
**LOOP 3**	**350G** **352T** **353G**	**350G** **353G**	**353G**	**282G** **285G**	**282G** **285G 287S** **288T**	-
**LOOP 4**	-	**366V** **367N**	**366V**	-	**299G**	**297M**

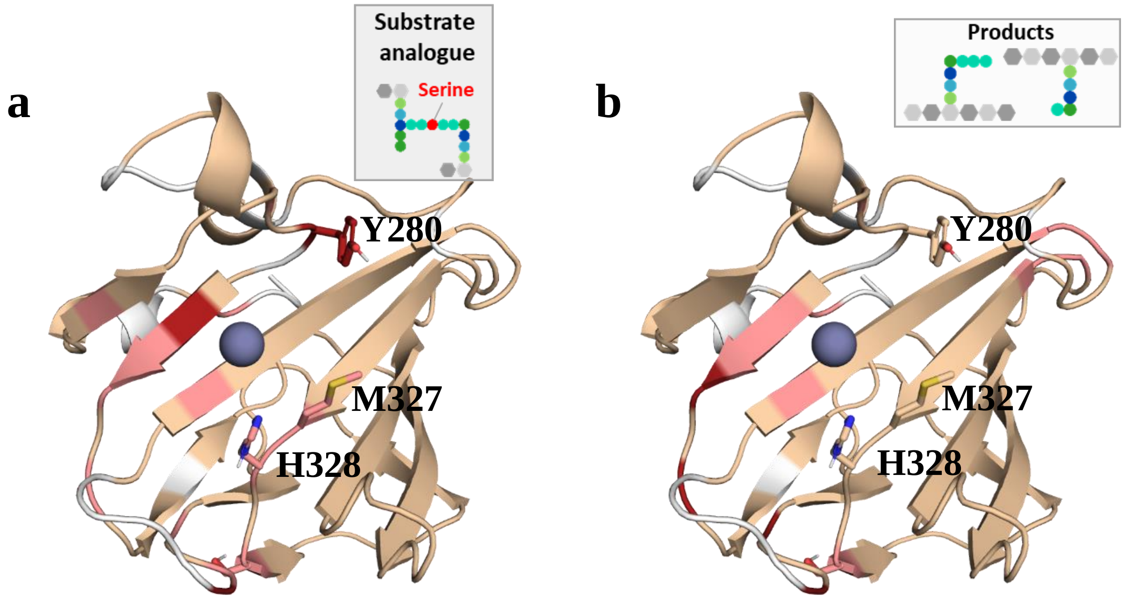

Supplementary Figure 6. Comparison of LssCD Zn^{2+}-binding groove perturbed regions with substrate analogue and M23 digestion product muropeptides. CSPs were recorded for titration experiment with substrate analogue (**a**) and reaction product (**b**), respectively. CSP superior to 1 standard deviation (> 0.25 ppm coloured in salmon) or 2 standard deviations (> 0.38 ppm for Lss coloured in crimson) are displayed on the cartoon representation of LssCD (PDB 5NMY). Side chains of the residues forming/in proximity of the active groove affected by titration with the substrate analogue were presented as sticks. The Zn atom is depicted as a blue-grey sphere.

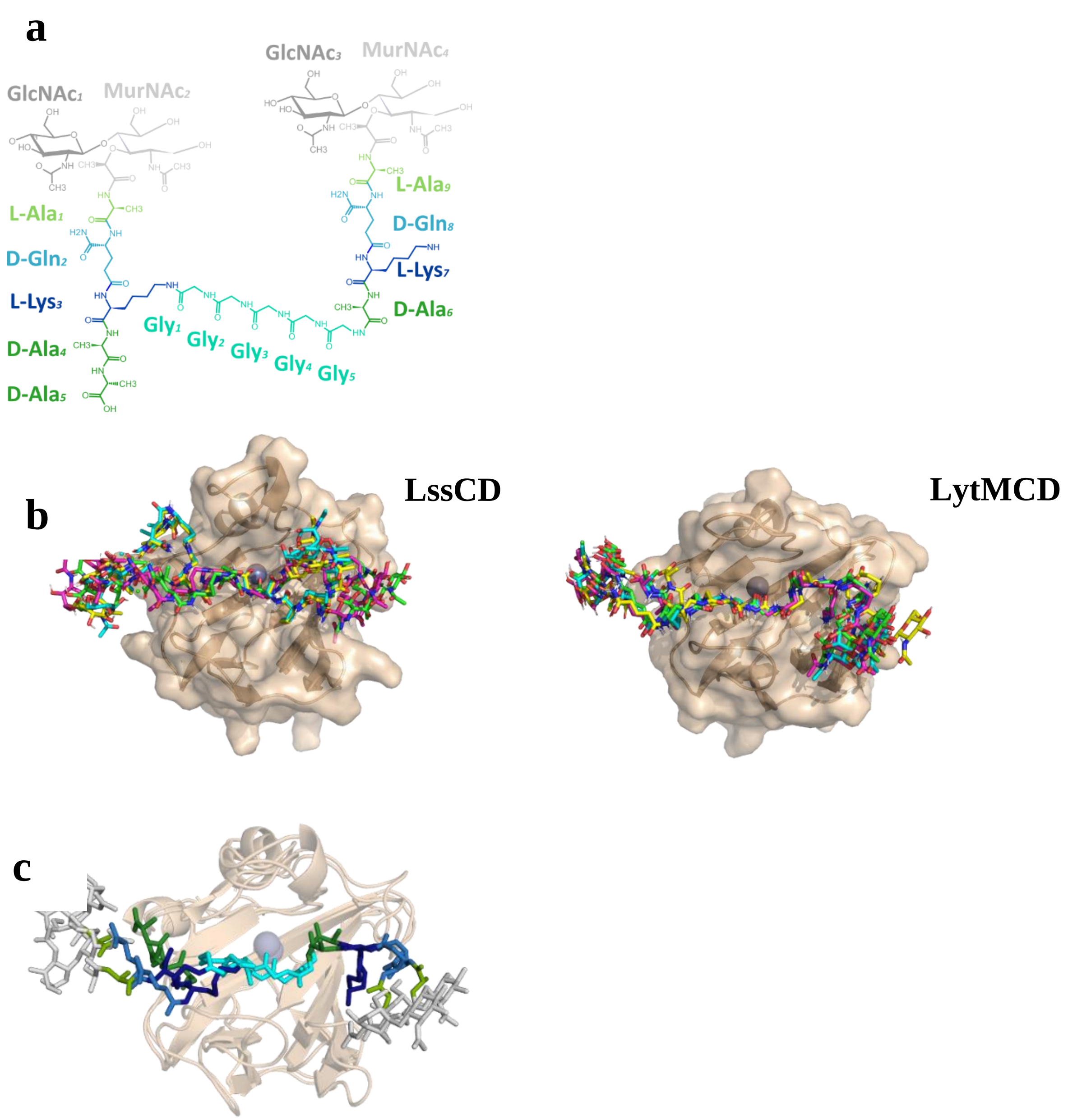

Supplementary Figure 7. HADDOCK modelling of the interaction between M23 enzymes and the substrate.

a, Chemical structure of the substrate molecule used for HADDOCK docking.

b, Four complexes of the lowest energy scores are presented. LssCD (left) and LytMCD (right) protein structures are presented as wheat ribbons with surface transparency in the same orientation with respect to the zinc active site. Only one of the four protein structures is presented for clarity. Ligands from the four superimposed complex structures are coloured differently. Substrate is shown in sticks representation, with N and O atoms in blue and red, respectively. Hydrogen atoms on muropeptides are omitted for clarity.

c, Overlap of the top two structures from the best convergence clusters obtained for LssCD (PDB ID: 5NMY) and LytMCD (PDB ID: 4ZYB). Ligand is coloured as in scheme on **Fig. 1a** in the main text.

Supplementary Table 4. Interacting residues forming H-bonds or van der Waals interactions with the ligand in HADDOCK models. β-sheet or loop localisation of the residues forming contacts with the ligand are indicated within parenthesis. Analysis of the contacts between the protein and the ligand was performed in the Liplot+ program. Their representatively is expressed as percentages of occurrence over the four best energy structures and is indicated in italics. Numbering of the ligand residues for the substrate and hexadimuropeptide models was included in **Fig. S8 and Fig. S11.**

		**LssCD**		**LytMCD**		
		**Substrate**	**Hexadimuropeptide**	**Substrate**	**Hexadimuropeptide**	
	**Ligand residues**	Dissacharide stem-peptides crosslinked by GGGGG peptides	Hexasaccharide stem-peptide with crosslinked by two GGGGG peptides	Dissacharide stem-peptides crosslinked by GGGGG peptides	Hexasaccharide stem-peptide with crosslinked by two GGGGG peptides	
**Cross-bridge**	**Gly$_3$**		-	**H328** (β-sheet) *75%*	-	-
	**Gly$_4$**		**H328** (β-sheet) *100%*	-	**Y204** (L1) *75%*	-
	**Gly$_5$**		-	-	**N286** (L3) *100%*	-
**Stem-peptide**	**D-Ala$_4$-D-Ala$_5$**		**K262** (β-sheet) *50%*	**K261** (β-sheet) *50%* **K262** (β-sheet) *50%*	**K196** (-) *100%* **Q199** (β-sheet) *75%*	**K196** (-) *80%* **H205**[d] (L1) *100%**
	**D-Ala$_6$**		**Q363** (β-sheet) *50%*	-	(backbone) **Y211** (L1) *100%*	**N303** (L4) *80%*
	**D-Gln$_8$**		-	**S305** (L2) *100%*	-	-
**Glycan strand**	**GlcNAc**		(backbone) **Y307**[a] (L2) *50%*	**Y307**[b] (L2) *100%* **S370**[c] (L4) *50%*	-	**Y239**[c] (L2) *80%*
	**MurNAc$_8$**		-	-	-	**Q304** (L4) *60%* **Y305** (L4) *60%*

Contacts with: [a] GlcNAc$_3$, [b] GlcNAc$_9$, [c] GlcNAc$_7$, [d] D-Ala$_{13}$-D-Ala$_{14}$
Abbreviations: L – loop.

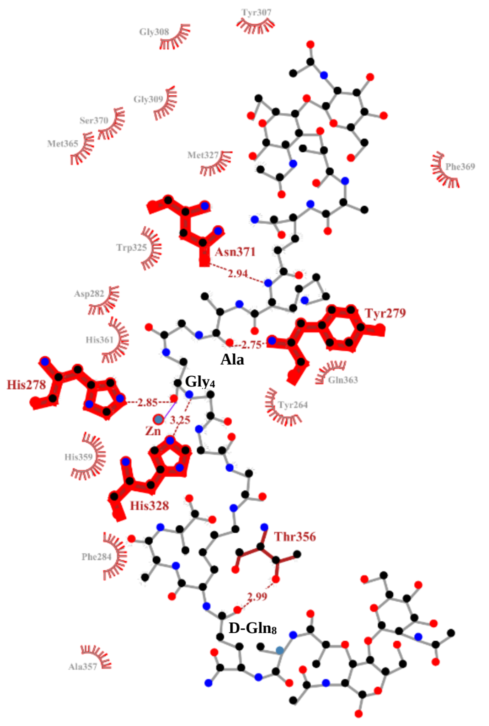

LssCD:substrate model, 1 best structure

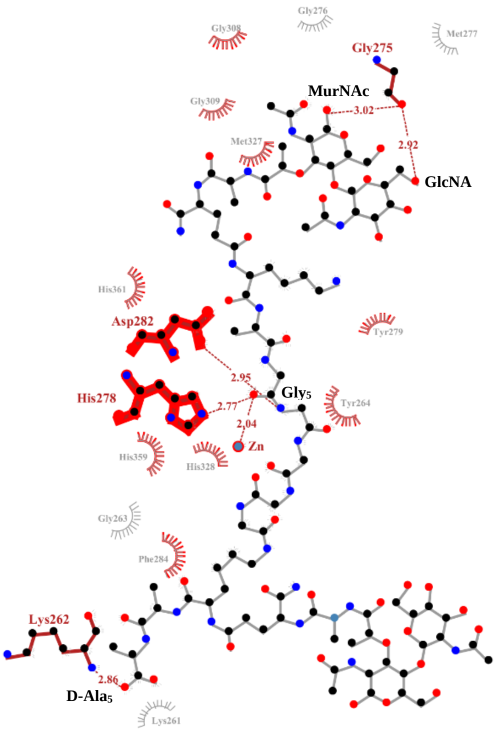

LssCD:substrate model, 2 best structure

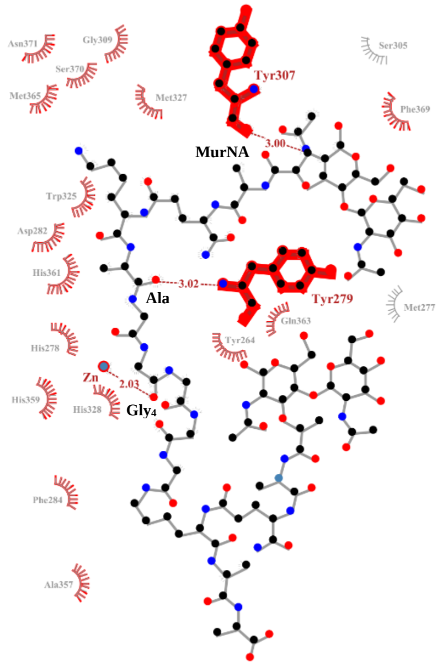

LssCD:substrate model, 3 best structure

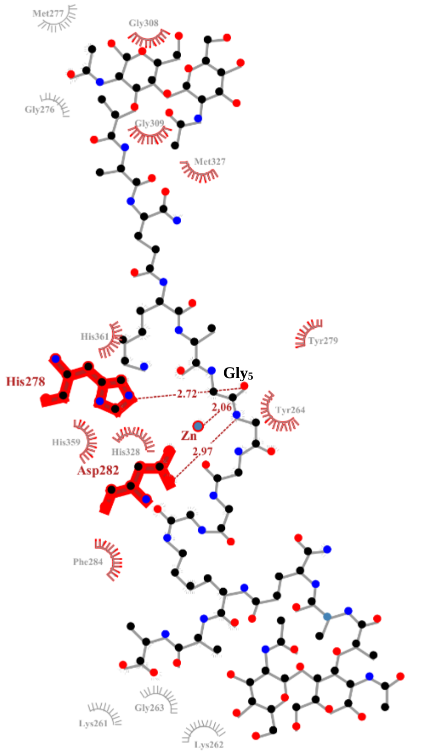

LssCD:substrate model, 4 best structure

Supplementary Figure 8. Schematic representation of the H-bond network (dashed lines) and van der Waals/hydrophobic interactions (semi-opened circle) in the four best structures of the HADDOCK LssCD:substrate model established with LigPlot+. The equivalent residues were marked with a red underlay. Equivalent residues engaged in hydrophobic interactions are shown in thicker red lines. H-bond forming ligand residues were named as presented on Fig. S7a. Key: red dot – oxygen atom, blue dot – nitrogen atom, black dot – carbon atom.

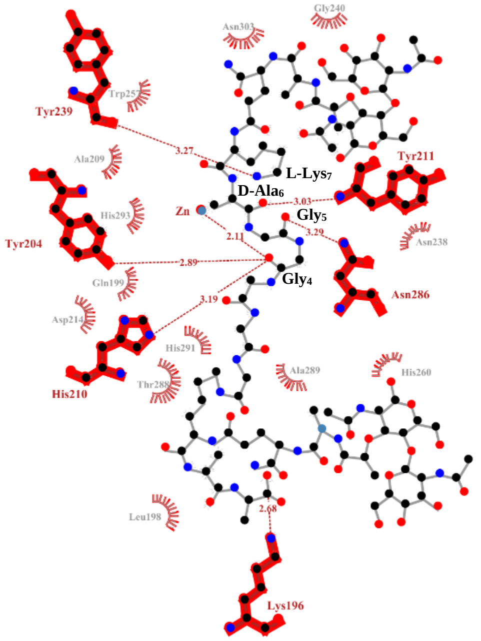

LytMCD:substrate model, 1st best structure

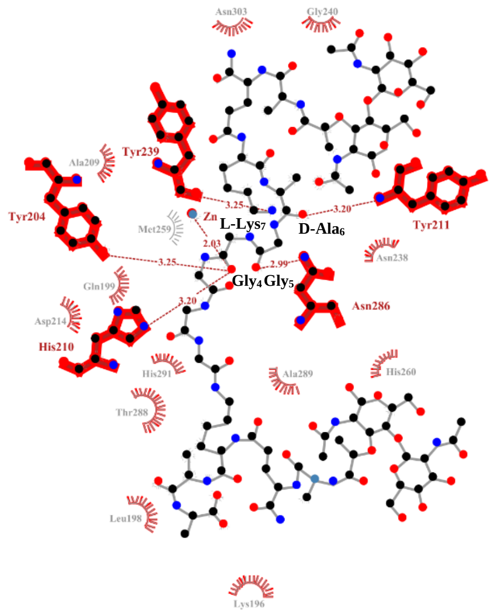

LytMCD:substrate model, 2nd best structure

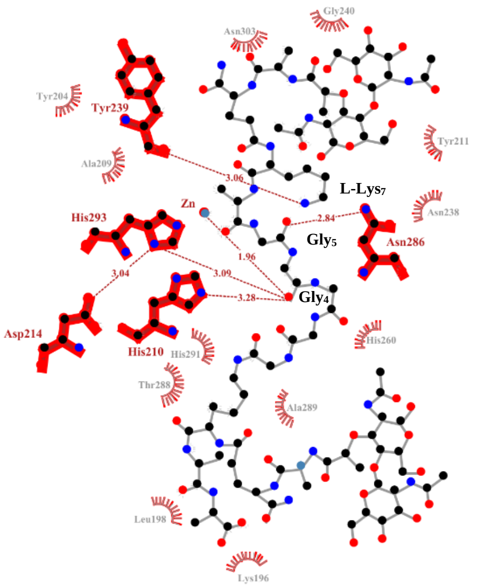

LytMCD:substrate model, 3rd best structure

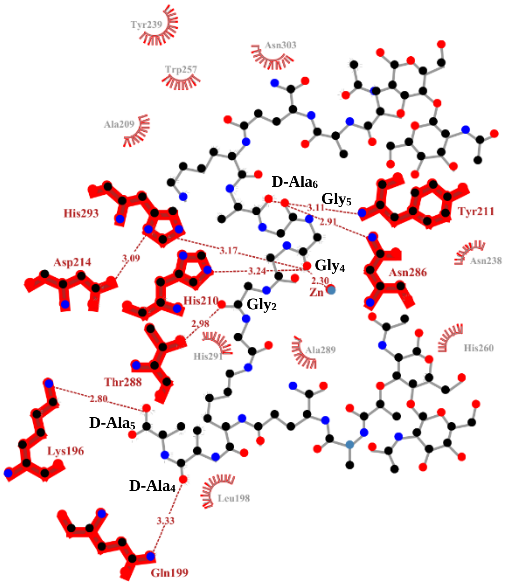

LytMCD:substrate model, 4th best structure

Supplementary Figure 9. Schematic representation of the H-bond network (dashed lines) and van der Waals/hydrophobic interactions (semi-opened circle) in the four best structures of the HADDOCK LytMCD:substrate model established with LigPlot+. The equivalent residues were marked with a red underlay. Equivalent residues engaged in hydrophobic interactions are shown in thicker red lines. H-bond forming ligand residues were named as presented on Fig. S7a. Key: red dot – oxygen atom, blue dot – nitrogen atom, black dot – carbon atom.

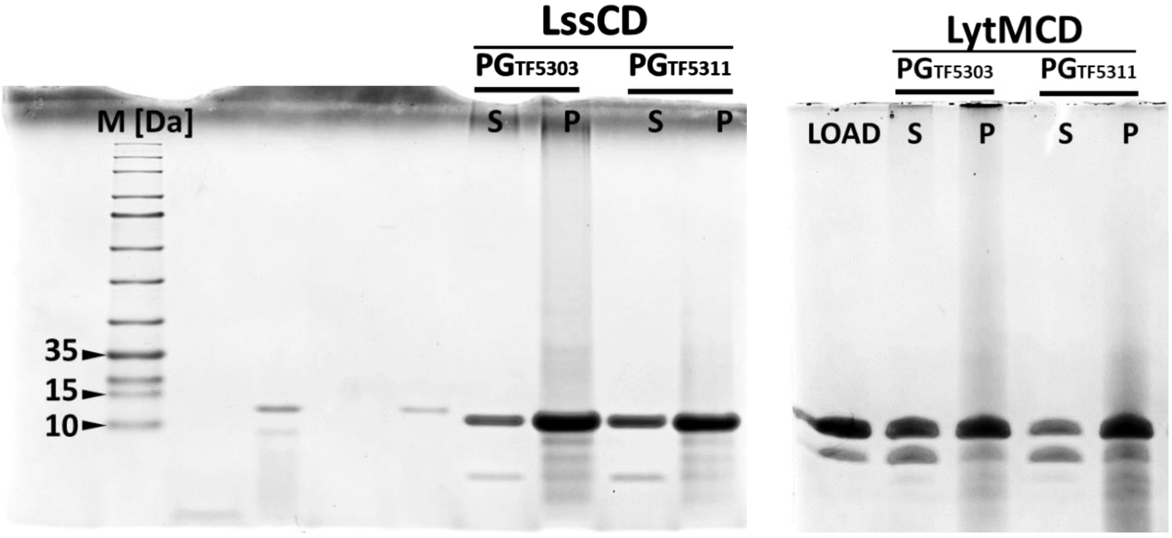

Supplementary Figure 10. Unprocessed original images of SDS-PAGE of LytMCD and LssCD pull-down to PG of *S. aureus* TF5303 (wild-type) and TF5311(serine mutant). Abbreviations: M – marker, S – supernatant (ubound fraction), P – pellet (bound fraction)**.**

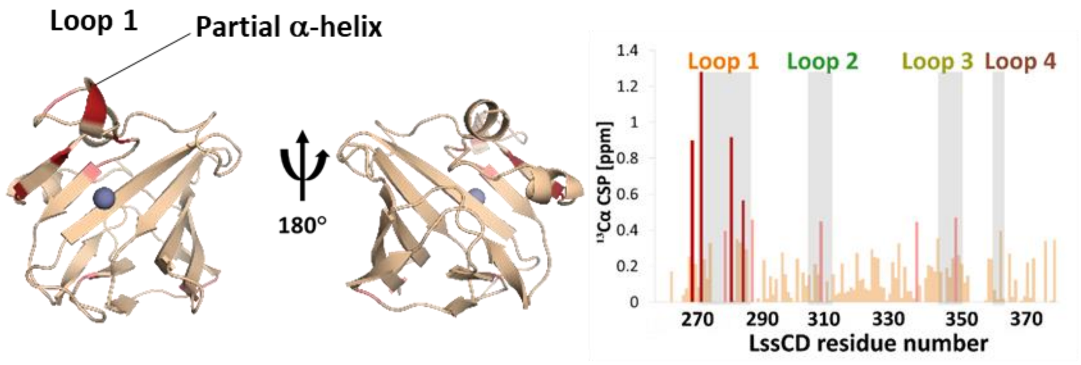

Supplementary Figure 11. Mapping of LssCD secondary structure distortions. Solution structure of LssCD (PDB: 5NMY) with marked ^{13}Cα CSPs superior to 1 and 2 standard deviations (> 0.37 ppm and > 0.56 ppm, respectively) coloured as in **Fig.** 5**b**. Partial a-helix is formed by 273I, 274N and 275G.

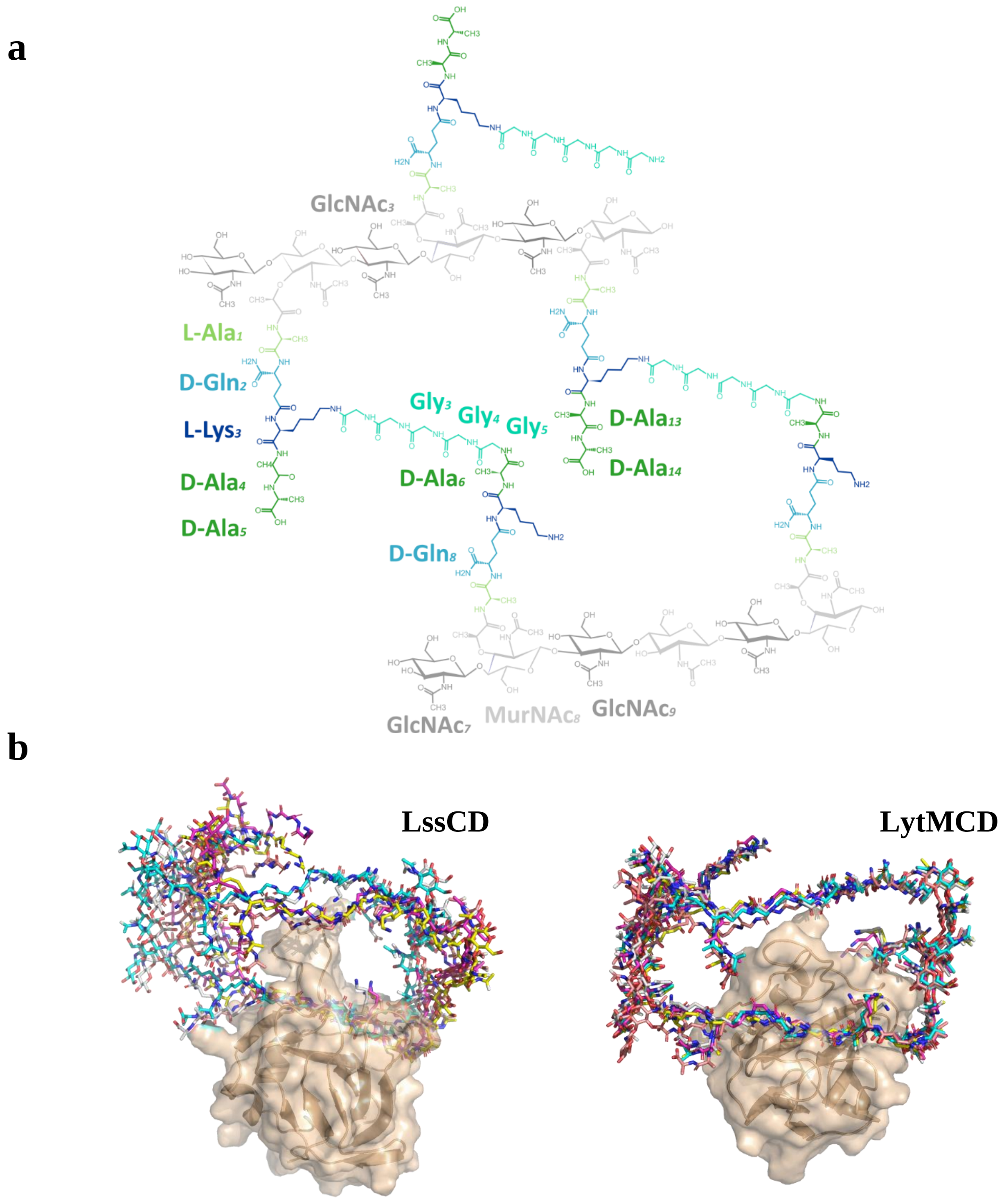

Supplementary Figure 12. HADDOCK modelling with the hexadimuropeptide.

a, Chemical structure of the substrate molecule used for HADDOCK docking. For clarity, only the residues which form contacts with proteins were marked.

b, HADDOCK modelling with the hexadimuropeptide. Five complexes of the lowest energy scores were selected. Structures were oriented ligand-wise to demonstrate differences in convergence between the two models. Structures are presented in the same orientation as in the **Fig. S7b**.

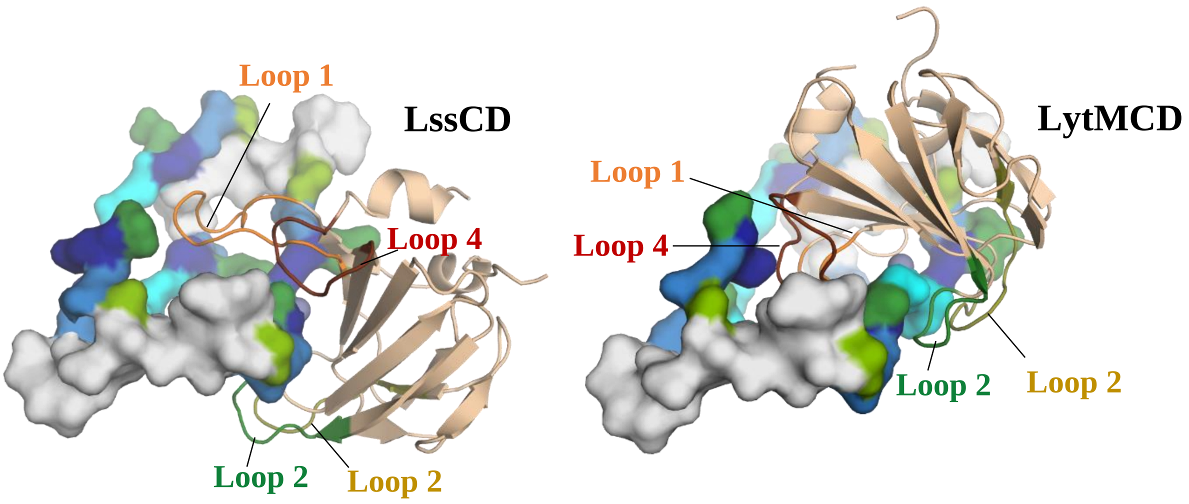

Supplementary Figure 13. Distinct positioning of loop 4 in LssCD and LytMCD. Models of the interaction with hexadimuropeptide were generated with HADDOCK. Ligand surface was coloured as in Fig. 1 in the main text. Enzymes were shown in cartoon representation. Loops were coloured as in Fig. 2c.

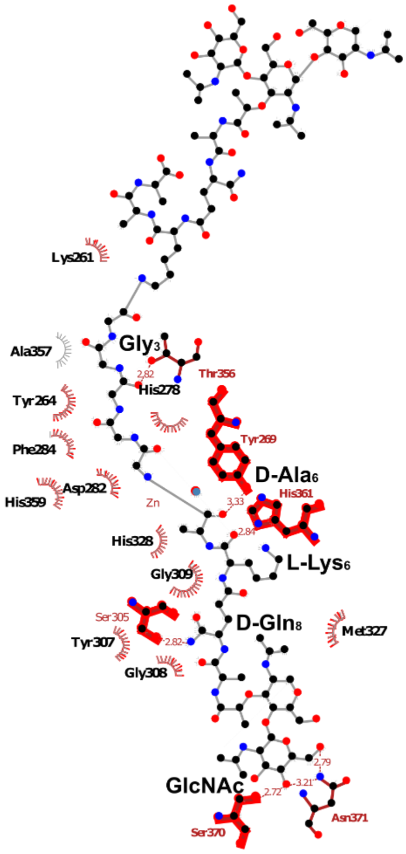

LssCD:hexadimuropeptide model
1st best structure

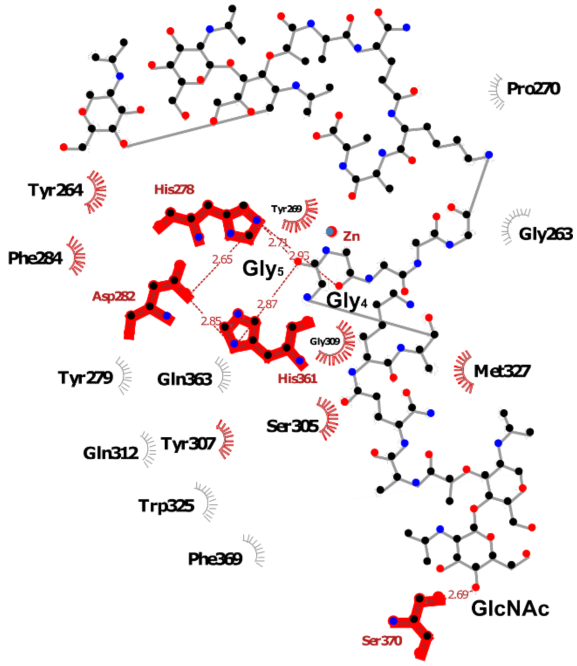

LssCD:hexadimuropeptide model
2nd best structure

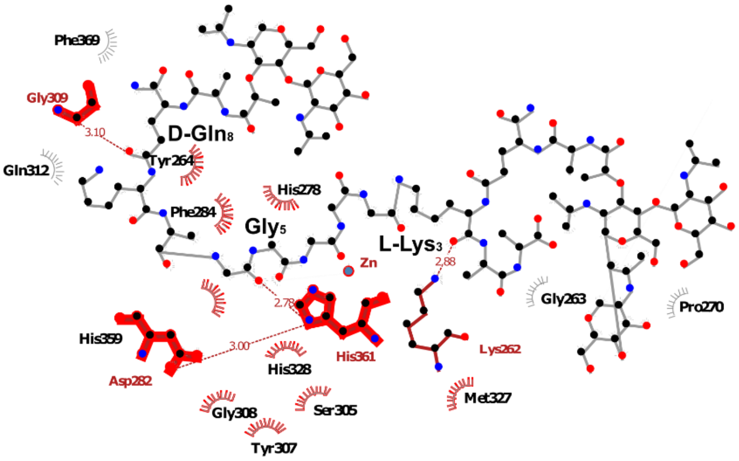

LssCD:hexadimuropeptide model
3rd best structure

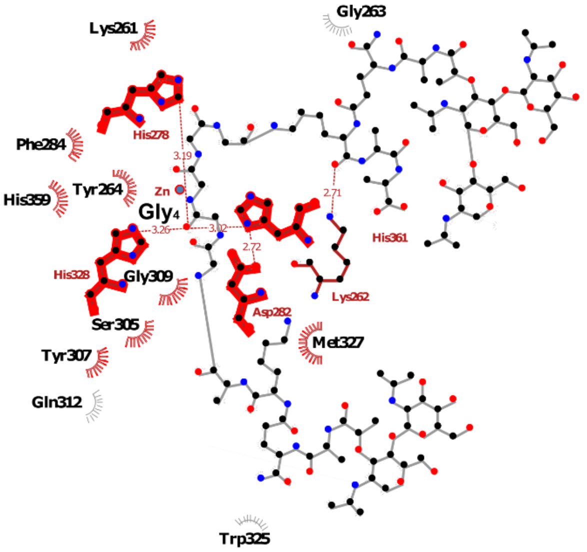

LssCD:hexadimuropeptide model
4th best structure

Supplementary Figure 14. Schematic representation of the H-bond network (dashed lines) and van der Waals/hydrophobic interactions (semi-opened circle) in the four best structures of the HADDOCK LssCD:hexadimuropeptide model established with LigPlot+. Due to technical limitations, only 20 ligand residues docked within the binding groove were included in the calculation. The equivalent residues were marked with a red underlay. Equivalent residues engaged in hydrophobic interactions are shown in thicker red lines. H-bond forming ligand residues were named as presented on Fig. S7a. Key: red dot – oxygen atom, blue dot – nitrogen atom, black dot – carbon atom.

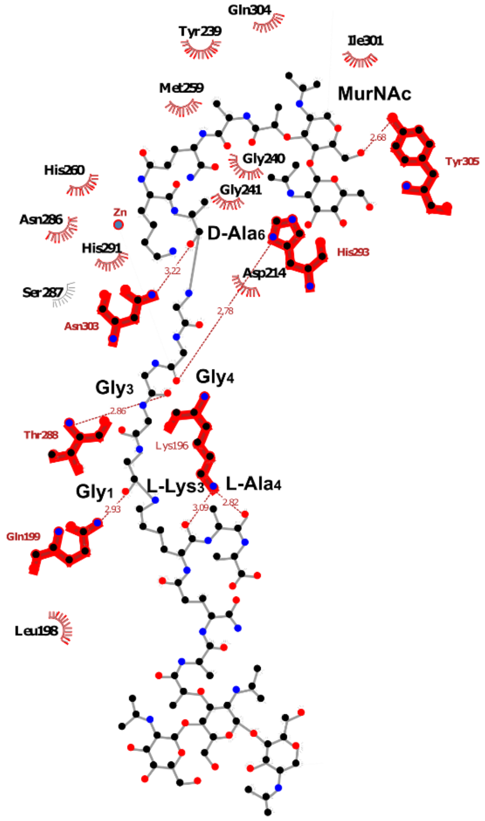

LytMCD:hexadimuropeptide model
1st best structure

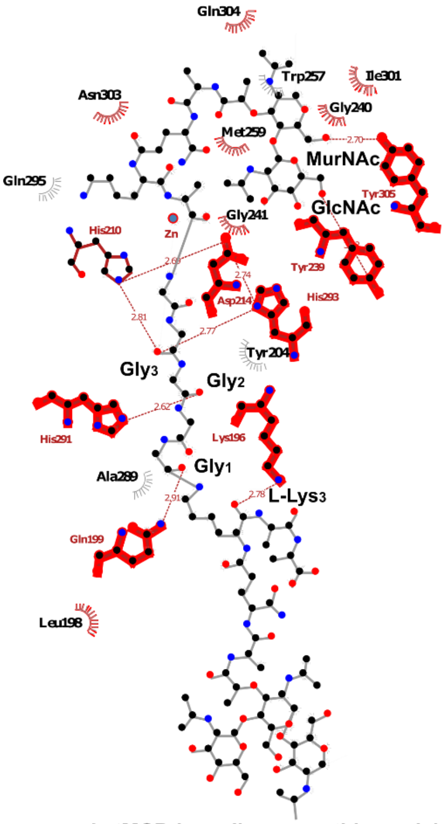

LytMCD:hexadimuropeptide model
2nd best structure

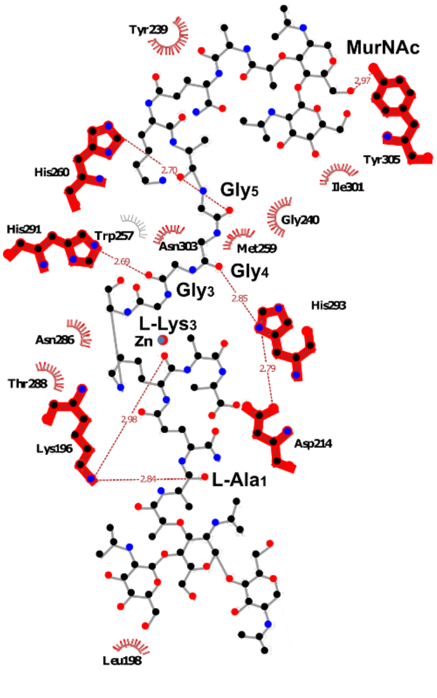

LytMCD:hexadimuropeptide model
3rd best structure

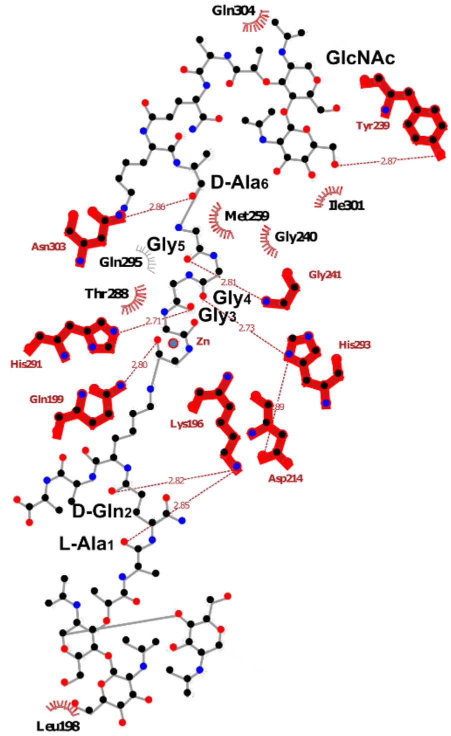

LytMCD:hexadimuropeptide model
4th best structure

Supplementary Figure 15. Schematic representation of the H-bond network (dashed lines) and van der Waals/hydrophobic interactions (semi-opened circle) in the four best structures of the HADDOCK LytMCD:hexadimuropeptide model established with LigPlot+. Due to technical limitations, only 20 ligand residues docked within the binding groove were included in the calculation. The equivalent residues were marked with a red underlay. Equivalent residues engaged in hydrophobic interactions are shown in thicker red lines. H-bond forming ligand residues were named as presented on Fig. S7a. Key: red dot – oxygen atom, blue dot – nitrogen atom, black dot – carbon atom.

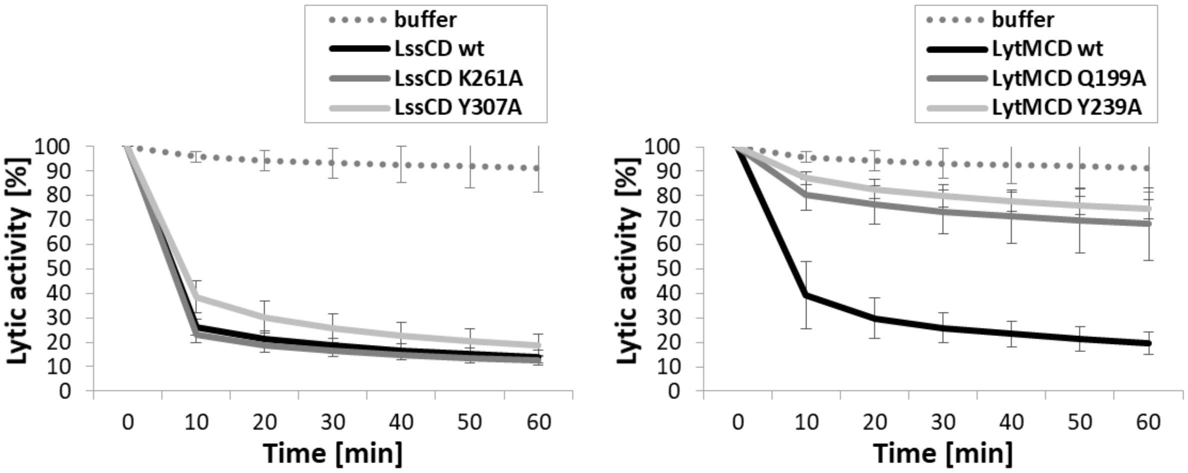

Supplementary Figure 16. Evaluation of the role in the lytic activity of LssCD and LytMCD amino acids forming contacts with stem-peptide and glycan chain in HADDOCK models. Turbidity reduction assay indicating lytic activity of wild-type enzymes and their variants against *S. aureus* TF5303 cells during 1 hour.

Supplementary Table 5. Primers, constructs and bacterial strains used in this study.

	**Name**	**Expressed protein**	**Reference**
**Constructs**	LssCD-pET15b	LssCD wt, no tag	[1]
	LssCD-K261A-pET15b	LssCD K261A, no tag	This study
	LssCD-Y307A-pET15b	LssCD Y307A, no tag	
	LytM-pET15b	LytMCD wt, no tag	[2]
	LytM-wt-pMSCG7	LytMCD wt, N-term His-Tag	This study
	LytM-K196A-pMSCG7	LytMCD K196A, N-term His-Tag	
	LytM-Q199A-pMSCG7	LytMCD Q199A, N-term His-Tag	
	LytM-Y239A-pMSCG7	LytMCD Y239A, N-term His-Tag	

	**Genetic product name**	**Sequence (5'→3')**	**Application**
	lytMCD_wt_HT	F:tacttccaatccaatgcccatgcgaaagacgcaagctggttaa<br>R:ttatccacttccaatgttatctactttgcaagtatgacgttgggtct	Fusion of LytM with His-tag
**Primers**	*lssCD_K261A*	R:gaataattacGCGaaaggatatggttacggtccttatc<br>F:accatatcctttCGCgtaattattcaaccattgtgctg	Site-Directed mutagenesis
	lssCD_Y307A	F:ggttggagtaatGCGggaggaggtaatcaaataggtc<br>R:attacctcctccCGCattactccaaccagcttcaactatttttc	
	lytMCD_K196A	F:gttaacaagtcgtGCGcaactacaaccatatggacaatatc<br>R:tggttgtagttgCGCacgacttgttaaccagcttgcgtc	
	lytMCD_Q199A	F:cgtaaacaactaGCGccatatggacaatatcacggtggt<br>R:ttgtccatatggCGCtagttgtttacgacttgttaaccag	
	lytMCD_Y239A	F:tggagtaacGCGggtggcggcaatcaagtaacg<br>R:attgccgccaccCGCgttactccaacctgcttgtac	
	- [primers specific for T7 promoter region]	F: taatacgactcactataggg<br>R: gctagttattgctcagcgg	Sequencing

	**Name**	**Application**	**Reference**
**Bacterial strains**	*E. coli* BL21(DE3)	Cloning	Laboratory strain
	S. aureus SH1000	PG labelling and purification	[3]
	S. aureus TF5303 (wild-type)	PG purification, lytic assays	[4]
	S. aureus TF5311 (serine containing cross-bridge)	PG purification	

Supplementary Table 6. HADDOCK restraints and HADDOCK Energy Statistics of the four dockings. Statistics are shown for the best cluster of each calculation. Abbreviations: Nstruc: Number of structures in the cluster, Evdw: Van der Waals intermolecular energy, Eelec: electrostatic intermolecular energy, Eair: distance restrains energy ambiguous.interactions restraints, BSA Buried Surface Area.

**Ligand model**	**Protein**	**Protein active residues**	**Unambiguous restraints**	**Ligand active residues**
**Substrate**	LssCD	267, 272, 274, 277, 279, 282, 283, 284, 286, 327, 328, 330, 350, 352, 353, 360, Zn	282, 361, 278 and Gly4 and Gly5;<br>Zn and 282, 361, 278	all
	LytMCD	197, 213, 217, 241, 267, 282, 285, 294, 295, 310, 316, Zn	214, 293, 210 and Gly4 and Gly5; Zn and 214, 293, 210	all
**Hexadi-muropeptide**	LssCD	253, 264, 272, 273, 275, 277, 278, 308, 313, 330, 332, 353, 366, Zn	-	all
	LytMCD	194, 217, 230, 231, 235, 239, 240, 241, 242, 246, 250, 289, 297, 311, 316, Zn	-	all

Ligand	Protein	Cluster	HADDOCK score	rmsd (Å)	Nstruc	Evdw	Eelec*	Eair*	BSA (Å)
**substrate**	LssCD	1	-71.3 ± 1.5	0.4 ± 0.2	198.0	-85.6 ± 33.8	-58.2 ± 18.7	24.1 ± 23.9	1282.2 ± 55.5
	LytMCD	1	-62.9 ± 1.2	0.3 ± 0.2	200.0	-50.3 ± 3.2	-115.0 ± 23.7	46.2 ± 11.7	1353.5 ± 16.0
**hexadi-muropeptide**	LssCD	2	-115.5 ± 12.3	2.3 ± 1.2	7.0	-41.8 ± 4.0	-342.2 ± 75.5	208.9 ± 101.8	1467.9 ± 98.0
	LytMCD	8	-136.7 ± 8.6	0.9 ± 0.5	6.0	-55.3 ± 7.8	-492.2 ± 31.0	314.5 ± 66.1	2169.0 ± 170.8

*(kcal/mol)

Supplementary references:

1. Jagielska, E., Chojnacka, O. & Sabała, I. LytM Fusion with SH3b-Like Domain Expands Its Activity to Physiological Conditions. *Microb Drug Resist* **22**, 461–469 (2016).
2. Odintsov, S. G., Sabala, I., Marcyjaniak, M. & Bochtler, M. Latent LytM at 1.3A resolution. *J Mol Biol* **335**, 775–785 (2004).
3. Horsburgh, M. J. *et al.* sigmaB modulates virulence determinant expression and stress resistance: characterization of a functional rsbU strain derived from Staphylococcus aureus 8325-4. *J Bacteriol* **184**, 5457–5467 (2002).
4. Sugai, M. *et al.* Purification and molecular characterization of glycylglycine endopeptidase produced by Staphylococcus capitis EPK1. *J Bacteriol* **179**, 1193–1202 (1997).
