# Supplementary material for: Staphylococcus aureus sacculus mediates activities of M23 hydrolases
Source: Nat Commun. 2023 Oct 23;14:6706. doi: 10.1038/s41467-023-42506-w (PMC10593780; doi:10.1038/s41467-023-42506-w)
Supplement: Supplementary file 3 — Description of Additional Supplementary Files [file 41467_2023_42506_MOESM3_ESM.pdf]

Description of Additional Supplementary Files

Supplementary Data 1. HADDOCK LssCD:substrate model. Top-score structure of the best convergence cluster obtained for LssCD HADDOCK models in complex with the substrate.

Supplementary Data 2. HADDOCK LytMCD:substrate model. Top-score structure of the best convergence cluster obtained for LytMCD HADDOCK models in complex with the substrate.

Supplementary Data 3. HADDOCK LssCD:hexadimuropeptide model. Top-score structure of the best convergence cluster obtained for LssCD HADDOCK models in complex with the hexadimuropeptide.

Supplementary Data 4. HADDOCK LytMCD: hexadimuropeptide model. Top-score structure of the best convergence cluster obtained for LytMCD HADDOCK models in complex with the hexadimuropeptide.
